# SV40 Polyomavirus Activates the Ras-MAPK Signaling Pathway for Vacuolization, Cell Death, and Virus Release

**DOI:** 10.3390/v12101128

**Published:** 2020-10-05

**Authors:** Nasim Motamedi, Xaver Sewald, Yong Luo, Walther Mothes, Daniel DiMaio

**Affiliations:** 1 Department of Genetics, Yale School of Medicine, P.O. Box 208005, New Haven, CT 06520-8005, USA; yluo2012@gmail.com; 2 Department of Microbial Pathogenesis, Yale School of Medicine, 295 Congress Avenue, New Haven, CT 06519-1418, USA; Sewald@mvp.lmu.de (X.S.); walther.mothes@yale.edu (W.M.); 3 Department of Molecular Biophysics & Biochemistry, Yale University, P.O. Box 208024, New Haven, CT 06520-8024, USA; 4 Department of Therapeutic Radiology, Yale School of Medicine, P.O. Box 208040, New Haven, CT 06520-8040, USA; 5 Yale Cancer Center, P.O. Box 208028, New Haven, CT 06520-8028, USA

**Keywords:** Simian virus 40, vacuoles, GM1, lytic infection, progressive multifocal leukoencephalopathy, PML, JCV, BKV, Ras

## Abstract

Polyomaviruses are a family of small, non-enveloped DNA viruses that can cause severe disease in immunosuppressed individuals. Studies with SV40, a well-studied model polyomavirus, have revealed the role of host proteins in polyomavirus entry and trafficking to the nucleus, in viral transcription and DNA replication, and in cell transformation. In contrast, little is known about host factors or cellular signaling pathways involved in the late steps of productive infection leading to release of progeny polyomaviruses. We previously showed that cytoplasmic vacuolization, a characteristic late cytopathic effect of SV40 infection, depends on the specific interaction between the major viral capsid protein VP1 and its cell surface ganglioside receptor GM1. Here, we show that, late during infection, SV40 activates a signaling cascade in permissive monkey CV-1 cells involving Ras, Rac1, MKK4, and JNK to stimulate SV40-specific cytoplasmic vacuolization and subsequent cell lysis and virus release. Inhibition of individual components of this signaling pathway inhibits vacuolization, lysis, and virus release, even though high-level intracellular virus replication occurs. Identification of this pathway for SV40-induced vacuolization and virus release provides new insights into the late steps of non-enveloped virus infection.

## 1. Introduction

The polyomaviruses are small, non-enveloped, double-stranded DNA tumor viruses that include pathogenic human viruses such as BK polyomavirus (BKPyV), JC polyomavirus (JCPyV), and Merkel Cell polyomavirus (MCPyV) as well as the extensively studied model viruses, murine polyomavirus and simian virus 40 (SV40). MCPyV, the most recently discovered human tumor virus, is responsible for most cases of Merkel cell carcinoma, a rare but aggressive form of skin cancer. BKPyV is associated with inflammation of the urogenital tract and nephropathy, which can result in organ loss in renal transplant patients as well as hemorrhagic cystitis in bone marrow transplant recipients (reviewed in [1]). JCPyV is the causative agent of progressive multifocal leukencephalopathy (PML), a rare but usually fatal central nervous system-demyelinating disease in immunocompromised individuals or patients receiving immunomodulatory monoclonal antibody treatment for various disorders [2]. JCPyV and BKPyV infections are common in the human population. These viruses are phylogenetically closely related to SV40, which can cause PML-like brain pathology in immunosuppressed monkeys [3,4]. Therefore, SV40 serves as a model to study human polyomavirus pathogenesis, including neurological disease. 

Productive polyomavirus infection of permissive cells can be divided into early and late phases. The early phase includes virus binding to the cell surface, entry of virus particles into the cell, and trafficking of the viral genome to the nucleus where viral gene expression and DNA replication occur. Polyomavirus entry is initiated by binding of the major capsid protein VP1 to carbohydrate motifs on cell surface molecules. In the case of SV40, the ganglioside GM1 serves as the cellular receptor for infection. After endocytosis, polyomaviruses are transported to the endoplasmic reticulum (ER), where host factors initiate the disassembly of capsids and translocation of the viral genome and residual capsid into the cytoplasm for transport into the nucleus [5,6]. Subsequent expression of the early viral proteins including large and small T antigens is followed by viral DNA replication, expression of the late viral proteins including VP1, and capsid assembly, which occurs primarily in the nucleus before cells are lysed and mature infectious virus particles are released.

The initial interaction of a variety of polyomaviruses with cells acutely induces transient cellular signaling that supports the early steps of infection. The JC virus induces ERK phosphorylation within minutes after receptor binding [7], which is required for the early stages of infection [8]. Within the first two hours of infection, murine polyomavirus induces the phosphoinositide 3′ kinase and Fak signaling pathways through binding of VP1 to gangliosides and alpha4-integrin [9,10,11]. Inhibition of these signaling events can inhibit the early steps of murine polyomavirus infection. This virus also induces a second, delayed wave of mitogenic signaling that depends on viral early gene expression [11]. Cell signaling also modulates productive SV40 infection [12]. Binding of SV40 to GM1 at the plasma membrane triggers activation of more than 50 different kinases regulating the early steps of SV40 infection including local activation of tyrosine kinases to reorganize actin filaments for caveolin-1- or lipid raft-dependent SV40 internalization [13]. 

In contrast to the early stages of polyomavirus infection, late events leading to the release of virus particles are poorly understood. Viral proteins have been reported to facilitate SV40 release from cells. The late protein VP4 has been reported to function as a viroporin with membrane-destabilizing properties that facilitates virus release, but these results have recently been challenged [14,15,16,17]. Furthermore, since VP4 is mostly found within the nucleus of infected cells, the mechanism leading to plasma membrane perforation and virus release is unclear. The minor capsid proteins VP2 and VP3 were also reported to support membrane permeabilization for virus release [14]. These proteins can insert into or disrupt membranes when ectopically overexpressed in prokaryotic as well as eukaryotic cells. 

SV40 infection of African green monkey cells leads to the appearance of characteristic cytoplasmic vacuoles late during infection, a phenomenon that led to the discovery of this virus in 1960 [18]. We showed that vacuolization is triggered by the interaction between VP1 and GM1 at the cell surface [19,20]. SV40-induced vacuolization typically occurs late in infection, but if large amounts of SV40 are added to cells, vacuoles can form acutely [19,21]. Virus replication is not required for vacuole formation, and purified VP1 pentamers are sufficient to induce vacuole formation. We hypothesized that the VP1–GM1 interaction triggers an as-yet-unidentified signaling cascade resulting in vacuolization [19]. 

Extensive cell vacuolization has also been observed in other experimental systems. Pore-forming toxins of various pathogens can induce the formation of cellular vacuoles and cell death [22,23]. Different types of intrinsic cell death programs, such as paraptosis and methuosis, are also associated with vacuole formation [22,24]. Cell signaling pathways including the Ras-MAPK pathway have been shown to contribute to vacuolization and nonapoptotic cell lysis in these processes [24,25]. However, cellular factors or signaling pathways have not been identified that are involved in vacuolization or other late events during SV40 infection. 

In this study, we investigate the mechanism by which SV40 infection results in efficient virus release. We show that activation of the Ras-Rac1-MKK4-JNK signaling pathway late during SV40 infection results in vacuolization and ultimately facilitates cell lysis and release of progeny virus. Understanding the mechanism of polyomavirus release may allow the identification of proteins and pathways that can potentially be exploited as specific antiviral drug targets for polyomaviruses and possibly other pathogenic, non-enveloped viruses.

## 2. Materials and Methods

### 2.1. Cells and Virus

CV-1 cells and SV40 776 virus DNA were purchased from American Type Culture Collection (ATCC). Cells were maintained in Dulbecco’s modified Eagle’s medium (DMEM) (Sigma Aldrich, St. Louis, MO, USA) supplemented with 10% fetal bovine serum (FBS), 10 mM L-glutamine, and 10 mM HEPES (pH 7.2) in 5% CO_2_ at 37 °C. SV40 was produced from SV40 776 in a bacterial vector backbone puc19 by excision, religation, and transfection into CV-1 cells. When significant cell death was observed, cell cultures were subjected to multiple rounds of freeze/thaw lysis. Cellular debris was removed by centrifugation at 1000 rpm for 5 min, and supernatants were filtered through 0.45-µm syringe filters, aliquoted, and stored at −80 °C. To produce higher titer virus stocks, fresh CV-1 cells were infected at an MOI of 0.5 and processed as described above.

### 2.2. Virus Titer Quantitation

To quantify infectious units of SV40, serial dilutions of virus preparation, tissue culture supernatant, or cell lysate were added to monolayers of 2 × 10^5^ CV-1 cells in six-well plates. After 24 h, CV-1 cells were trypsinized, fixed, and permeabilized in methanol or with 4% PFA/0.5% Triton X-100 before being subjected to immunofluorescence staining for large T antigen and flow cytometry. In a typical infection with wildtype SV40, at 48 h.p.i., there is approximately 5- to 9-fold more virus in the supernatant than in the cell lysate.

### 2.3. Immunoblots

CV-1 cells were seeded at 2 × 10^5^ in six-well plates and infected with SV40 on the following day at an MOI of 10. At indicated times p.i., cells were harvested by lysis in lysis buffer (2% Triton X-100, 0.5% Na-deoxycholate, 150 mM NaCl, 25 mM Tris, 5 mM EDTA, halt protease, and phosphatase inhibitors (Thermo Scientific, Waltham, MA, USA )). Extracts were suspended in 5 × Laemmli buffer, subjected to sonication, and boiled. Equal sample volumes were loaded on sodium dodecyl sulfate polyacrylamide gel electrophoresis (SDS-PAGE) for protein separation. Proteins were then transferred to 0.2-μm polyvinylidene difluoride (PVDF) membranes in Tris/glycine transfer buffer (25 mM Tris, 192 mM glycine, and 20% methanol) for 2 h at 100 V. Membranes were blocked in 5% bovine serum albumin (BSA) or non-fat dry milk in Tris-buffered saline with Tween-20 (TBST) (10 mM Tris-HCl, pH 7.4, 167 mM NaCl, and 1% Tween-20) for 1 h and incubated overnight at 4 °C with indicated antibodies in 5% BSA/TBST for phosphorylated targets or in 5% non-fat dry milk/TBST for all others. Blots were washed in TBST and incubated for 1 h at room temperature with HRP-conjugated donkey anti-mouse/rabbit/goat (Jackson ImmunoResearch, Philadelphia, PA, USA) in 5% non-fat dry milk/TBST. After washing with TBST, blots were visualized by enhanced chemiluminescence (SuperSignal West Pico/Femto Chemiluminescent Substrate, Thermo Scientific). The following primary antibodies were used for immunoblotting: anti-ß-actin (Abcam, #ab8227), anti-p-ERK (Cell Signaling Technology, CST, #4370), anti-p-JNK (CST, #4668), anti-p-p38 (CST, #4511), anti-p-MKK4 (CST, #9156), anti-ERK (CST, #4695), anti-JNK (CST, #9252), anti-p38 (CST, #8690), anti-p-ATF2 (CST, #9225), anti-p-cJun (CST, #3270), anti-large T antigen (PAb, #108), and anti-VP1 (PAb, #597).

### 2.4. Vacuolization Assay

CV-1 cells were plated at a density of 2 × 10^5^ cells per well on six-well plates and infected with SV40 on the following day at an MOI of 10. At indicated time points, vacuolization was assessed and documented by bright-field microscopy. Only cells containing vacuoles with a diameter larger than 5 µm were counted positive. Mock-treated cells were initially used as a reference to differentiate membrane vesicles of the endosomal system from SV40-induced vacuoles. A minimum of 200 cells per sample were counted under blinded conditions.

### 2.5. Chemical Inhibitor Treatment

CV-1 cells were plated at a density of 2 × 10^5^ on six-well plates. On the following day, cells were infected with SV40 at an MOI of 10. Twelve h.p.i. or at the time of infection (0 h.p.i.), chemical inhibitors were added at a concentration of 25 µM to the infected and mock-treated CV-1 cells. Inhibitor treatment was maintained until the end of the experiment. The following chemical inhibitors were used in this study: Selumetinib (ERK pathway inhibitor, inhibits MEK1), EHT 1864 (Rac inhibitor), SB203580 (p38 inhibitor), and SP600125 (JNK inhibitor). All inhibitors were purchased from Selleckchem (Houston, TX, USA).

### 2.6. qRT-PCR

For quantitative PCR, total RNA was extracted from 2 × 10^5^ cells using the RNEasy Mini Kit (Qiagen, Hilden, Germany) and a maximum of 1 µg cDNA was transcribed with the iScript™ cDNA Synthesis Kit (BioRad, CA, USA). The relative expression levels were assessed in triplicate on a single-color detection system (BioRad CFX Connect Real-Time PCR Detection System) with the iTaq™ universal SYBR Green supermix (BioRad, CA, USA). Genes and primers used for qPCR were as follows: *GAPDH* forward (TGGTATCGTGGAAGGACTCA), *GAPDH* reverse (CCAGTAGAGGCAGGGATGAT), *MKK4* forward (TGAAAAGGCACAAAGTAAACGCA), and *MKK4* reverse (CCCAGTGTTGTTCAGGGGAG).

### 2.7. Dextran and BODIPY-GM1 Treatment

CV-1 cells were plated at a density of 2 × 10^4^ on Lab-Tek II chambered coverglass slides (Nunc) and infected with SV40 at an MOI of 100 on the following day. At 47 h.p.i., cells were incubated for 1 h with fluorescent markers. For dextran uptake assays, the cell culture medium was supplemented with 0.25 mg/mL of 3 kDa Dextran conjugated with Alexa Fluor 488 (Dextran-AF488, Life Technologies, USA). For labeling with fluorescent GM1, 5 µM BODIPY FL C5-GM1 (Molecular Probes) was added to the cell culture medium. At 48 h.p.i., cells were thoroughly washed and fresh cell culture medium was added for imaging. Cells were imaged at 50 h.p.i. on a Nikon TE2000 spinning disk confocal microscope driven by the Volocity software package (Perkin Elmer, Hopkinton, MA, USA).

### 2.8. Immunofluorescence

CV-1 cells were plated at a density of 3 × 10^4^ on Millicell EZ Slide four-well glass slides (Millipore, USA). On the following day, cells were infected with SV40 at an MOI of 100. After 48 h, cells were fixed with 4% PFA, permeabilized with 0.5% Triton X-100, and immunostained or treated with 0.5 µg/mL Alexa Fluor 488 (AF488)-conjugated CtxB (Molecular Probes) to stain GM1. The following primary antibodies were used for immunofluorescence staining: anti-VP1 (Abcam, #ab53977), anti-EEA1 (CST, #C45B10), anti-Rab7 (CST, #D95F2), and anti-BiP (Abcam, #ab108615). The secondary antibodies donkey anti-mouse/anti-rabbit conjugated to Alexa Fluor-488 or -568 (Life Technologies, USA) were used. Stained samples were embedded in ProLong Gold (Invitrogen, Waltham, MA, USA), and data were acquired on a spinning disk confocal microscope (Nikon, Japan). Images were analyzed using Volocity software (PerkinElmer).

For experiments involving Ras expression, 2 × 10^5^ CV-1 cells were plated on coverslips in six-well plates. Cells were transfected with wildtype (WT) or dominant-negative (DN) mEGFP-HRas using FuGENE6 (Promega, Madison, WI, USA) transfection reagent. On the following day, cells were infected with SV40 at an MOI of 10. At 48 h.p.i., the cells were fixed and immunostained with a primary antibody against anti-VP1 (Abcam, Cambridge, UK) and the secondary antibody anti-rabbit Alexa Fluor 568. Stained samples were embedded in ProLong Gold (Invitrogen, MA, USA), and data were acquired on a spinning disk confocal microscope (Nikon, Japan). Images were analyzed using Volocity software (PerkinElmer, MA, USA). The plasmids encoding WT mEGFP-HRas (Plasmid #18662) and DN mEGFP-HRas S17N (Plasmid #18665) were purchased from Addgene (Watertown, Massachusetts, USA).

### 2.9. Live-Cell Microscopy

CV-1 cells were plated at a density of 1.5 × 10^4^ on Lab-Tek II chambered coverglass slides (Nunc). On the following day, cells were infected with SV40 at an MOI of 100. At 20 h.p.i., cells were co-transfected with plasmids encoding Lamp1-RFP and YFP-Rab5 using FuGENE6 (Promega, WI, USA) transfection reagent. Time-lapse microscopy started at 40 h.p.i. Images were acquired every 15 min on a Nikon spinning disk confocal microscope with Nikon perfect focus system and a LiveCell environmental chamber (Pathology Devices, Westminster, CA, USA). Volocity software (PerkinElmer, MA, USA) and ImageJ were used for 4D image analysis.

### 2.10. Generation of MKK4 Knockdown CV-1 Cells with shRNA

Three different shRNAs to *MKK4* were generated using the MISSION library (Sigma, USA) and a lentiviral system consisting of pRSV, pMDL, and pVSV-G. Briefly, the virus was produced by transfection of HEK293 cells with the transfer and packaging vectors using FuGENE6 (Promega, WI, USA). At 24 and 48 h.p.i., the virus-containing supernatant was filtered using a 0.45-µm nylon membrane filter and stored at −80 °C. Pooled virus preparations were used for CV-1 target cell transduction. At about 24 h.p.i., puromycin treatment was started and maintained until the end of experiments. *MKK4* knockdown was verified by qPCR.

The following *MKK4* shRNAs were used in this study:

A12: TRCN0000001390

(CCGGCTTCTTATGGATTTGGATGTACTCGAGTACATCCAAATCCATAAGAAGTTTTT)

B1: TRCN0000001391

(CCGGGATGTATGAAGAACGTGCCGTCTCGAGACGGCACGTTCTTCATACATCTTTTT)

B3: TRCN0000001393

(CCGGGATATGATGTCCGCTCTGATGCTCGAGCATCAGAGCGGACATCATATCTTTTT)

Scrambled shRNA: SHC002

(CCGGCAACAAGATGAAGAGCACCAACTCGAGTTGGTGCTCTTCATCTTGTTGTTTTT)

### 2.11. Flow Cytometry

Flow cytometry was done as previously described [26]. Briefly, to stain for intracellular VP1 and large T antigen, cells were fixed and permeabilized with methanol or 4% PFA/0.5% Triton X-100 and subsequently stained with the primary antibodies PAb 597 and PAb108 against VP1 and large T antigen, respectively. Alexa Fluor 488-labeled donkey anti-mouse antibody (Jackson ImmunoResearch, PA, USA) or goat anti-mouse APC were used as secondary antibodies. Data were acquired on an AccuriC6 or FACS Calibur flow cytometer (BD Biosciences, USA) and analyzed with FlowJo software (Treestar, OR, USA).

### 2.12. LDH Release Assay

Lactate dehydrogenase (LDH) release into the cell culture supernatant was quantified using the CytoTox 96 nonradioactive cytotoxicity assay (Promega, WI, USA) according to the manufacturer’s instructions. Raw data were collected on a spectrophotometer at 490 nm. Values were calculated as follows: OD (infected sample) − OD (mock-infected sample)/OD (infected biological control) − OD (mock-infected biological control).

### 2.13. Data Analysis

All data were primarily processed in Microsoft Excel. Statistical analysis and graph production were performed using Prism software (Graphpad, CA, USA). For statistical analysis of data, an unpaired *t*-test was used.

## 3. Results

### 3.1. Phenotypic Characterization of SV40-Induced Vacuoles

**Figure 1:**
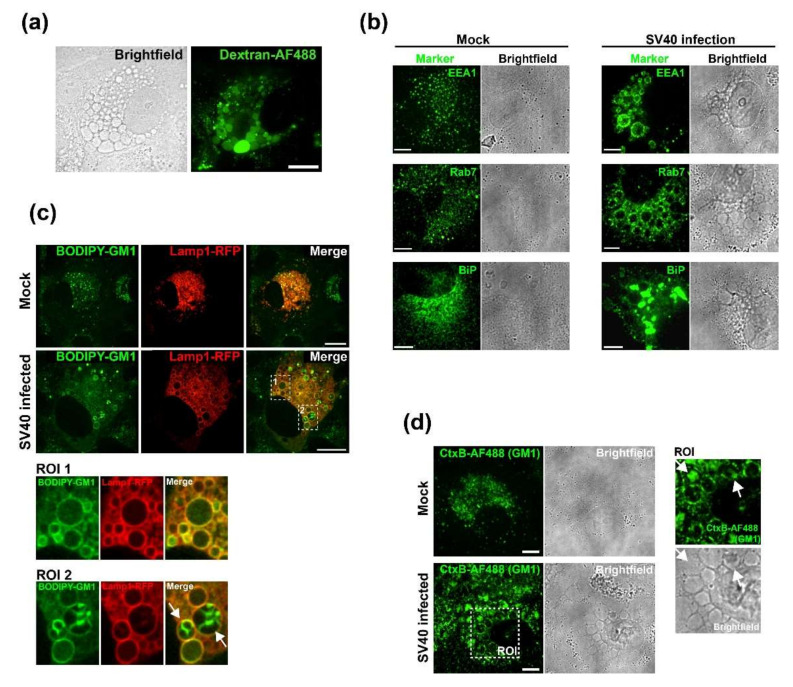
Characterization of SV40-induced vacuoles: (**a**) corresponding representative fluorescence and bright-field images of SV40-infected CV-1 cells 48 h.p.i. after incubation with medium containing fluorescent Dextran-AF488 (green), at scale bar 20 µm; (**b**) immunostaining and brightfield images of mock-infected and SV40-infected CV-1 cells 48 h.p.i. with antibodies recognizing markers of early endosome (EEA1), late (Rab7) endosome, and the endoplasmic reticulum (BiP), as indicated, at scale bar 10 µm; and (**c**) fluorescence microscopy images of mock-infected and SV40-infected CV-1 cells pulse-labeled 48 h.p.i. with fluorescent GM1 (BODIPY-GM1, green). CV-1 cells expressing Lamp1-RFP (red) were visualized by confocal microscopy. Arrows in regions of interest (ROIs) 1 and 2 highlight vacuoles in infected cells showing BODIPY-GM1 in the limiting membranes and interior of Lamp1-positive vacuoles, respectively. Single planes of z-stacks are shown at scale bar, 20 µm. (**d**) Fluorescence confocal microscopy and brightfield images of endogenous GM1 stained with fluorescent cholera toxin B (CtxB-AF488) (green) in mock-infected and SV40-infected CV-1 cells 48 h.p.i.: single planes of z-stacks are shown. Arrows in ROIs depict CtxB-staining of vacuole membranes and intravacuolar GM1 in infected cells at scale bar 10 µm.

We recently demonstrated that SV40-induced vacuole formation is triggered by binding of oligomeric VP1 to GM1 [19]. Vacuolization typically occurs late during infection, but a detailed analysis of vacuole formation and its consequences for SV40 infection is lacking. We conducted physiological experiments, immune staining, and live cell imaging to better characterize SV40-induced vacuoles. At 48 h.p.i., vacuoles present displayed an endocytic character as demonstrated by the rapid uptake of fluorescent low-molecular weight dextran (3 kDa Dextran-AF488) from the culture medium (Figure 1A). The endosomal nature of vacuoles was supported by immunostaining-infected cells with antibodies against the early and late endosomal proteins EEA1 and Rab7, respectively, which revealed staining distributed around the circumference of vacuoles (Figure 1B), presumably indicating the presence of these proteins at the vacuolar membrane. Although strong aggregation of the endoplasmic reticulum (ER) protein BiP was observed after infection compared to mock-treated cells, BiP did not associate with vacuole membranes, suggesting that SV40-induced vacuoles are not composed of ER membranes (Figure 1B).

Because vacuole formation is triggered by the interaction between VP1 and GM1, we assessed whether GM1 is present in vacuoles. To determine the localization of GM1 during SV40-induced vacuole formation late during infection, infected and mock-infected CV-1 cells expressing fluorescently-tagged Lamp1-RFP fusion protein were treated with fluorescently labelled GM1 (BODIPY-GM1) and analyzed by confocal microscopy at 48 h.p.i. In contrast to mock-infected cells where GM1 displayed diffuse punctate staining, in infected cells, GM1 (as well as the late endosomal/lysosomal marker Lamp1) was present at the limiting membrane of vacuoles (Figure 1C). Endogenous GM1 displayed a similar distribution in vacuolar membranes as assessed by staining with fluorescent cholera toxin B (CtxB-AF488) which, like VP1, binds to GM1 (Figure 1D). In addition, strong patches of GM1 were present within some vacuoles (arrows) (Figure 1C, D, regions of interest (ROI)), suggesting the existence of complex vesicular structures containing GM1 in infected cells during the late phase of infection.

**Figure 2:**
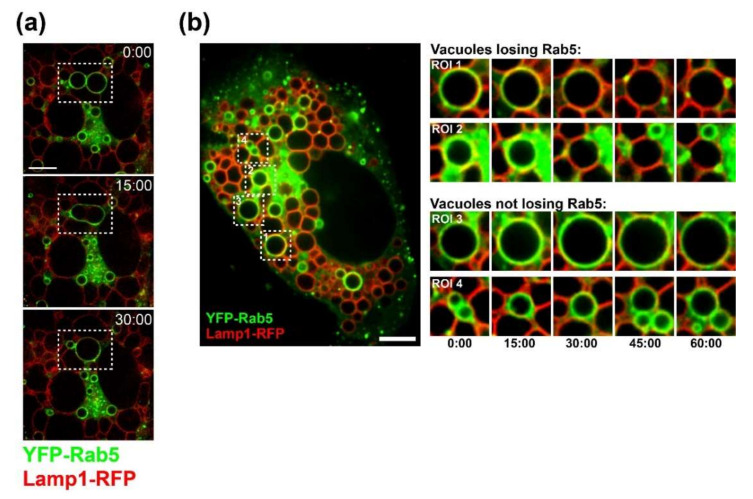
Dynamic vacuole formation: (**a**) image sequence (top to bottom) from a time-lapse movie (Movie S1) showing fusion of YFP-Rab5-positive vacuoles in an SV40-infected CV-1 cell. Lamp1-RFP is shown in red. The boxes outline two YFP-Rab5 vacuoles that fuse. Numbers in this and panel B show time in minutes. Scale bar, 5µm; (**b**) Overview image and time series of four regions of interest (ROI 1 to 4) from time-lapse movie S2 showing YFP-Rab5 (green) dynamics on SV40-induced vacuoles (Movie S2): ROIs 1 and 2 show vacuoles that lose YFP-Rab5 fluorescence, and ROIs 3 and 4 show vacuoles with stable YFP-Rab5 fluorescence. Lamp1-RFP is shown in red. Scale bar, 10µm.

To visualize the dynamics of vacuole formation and maturation, we conducted live-cell imaging using spinning-disk confocal microscopy of SV40-infected CV-1 cells transiently co-expressing fluorescently tagged versions of the early endosome marker YFP-Rab5 and the lysosome marker Lamp1-RFP. Both marker proteins localized to vacuole membranes (Figure 2). Fusion of Rab5-positive vacuoles was observed, indicating that the formation of large vacuoles was the result of fusion, not osmotic vesicle swelling (Figure 2A; Movie S1). In addition, some Rab5-positive vacuoles matured into vacuoles containing Lamp1 (with the loss of Rab5 in some cases) (Figure 2B; Movie S2), indicating that a dynamic endosomal system was involved in vacuole formation.

### 3.2. Vacuole Formation Requires Ras Activity

**Figure 3:**
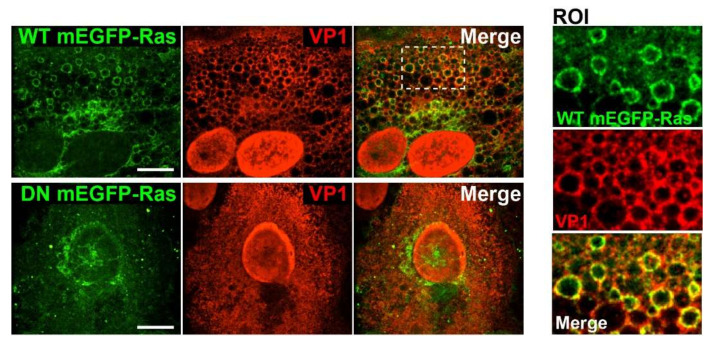
Ras signaling is required for vacuole formation. Fluorescence confocal microscopy images of SV40-infected CV-1 cells expressing wildtype (WT) or dominant-negative (DN) mEGFP-HRas (green): at forty-eight h.p.i., the localization of SV40 VP1 (red) was determined by immunostaining. The ROI depicts WT mEGFP-HRas accumulation at VP1-positive vacuoles. Single planes of z-stacks are shown. Scale bar, 10µm.

Expression of activated Ras can lead to vacuole formation in glioblastoma and other cancer cell lines [24]. To test for a role of Ras in SV40-induced cell vacuolization, we used a dominant-negative (DN) form of Harvey-Ras (HRas S17N), which inhibits the activity of all three Ras isoforms (H-, K-, and N-Ras) in COS-7 cells, a cell line derived from CV-1 cells used in this study. [27]. Plasmids expressing wildtype (WT) or DN versions of H-Ras, both fused to mEGFP, were transfected into CV-1 cells, which were infected 12 h later with SV40. At 48 h.p.i., wildtype mEGFP-HRas co-localized to the membranes of vacuoles with VP1 but did not affect vacuolization as assessed by VP1 immunostaining and fluorescence microscopy (Figure 3, upper panels and ROI). In contrast, expression of DN mEGFP-HRas S17N potently blocked SV40-induced vacuolization, even though VP1 was abundantly expressed (Figure 3, lower panels). Flow cytometry of large T antigen expression in CV-1 cells gated for mEGFP-Ras expression revealed no difference in SV40 infection efficiency in cells expressing wildtype compared to DN mEGFP-HRas (Supplemental Figure S1). These results indicate that Ras signaling is required for SV40-induced vacuole formation but not for SV40 infection.

### 3.3. Vacuolization Precedes Cell Lysis and SV40 Release

**Figure 4:**
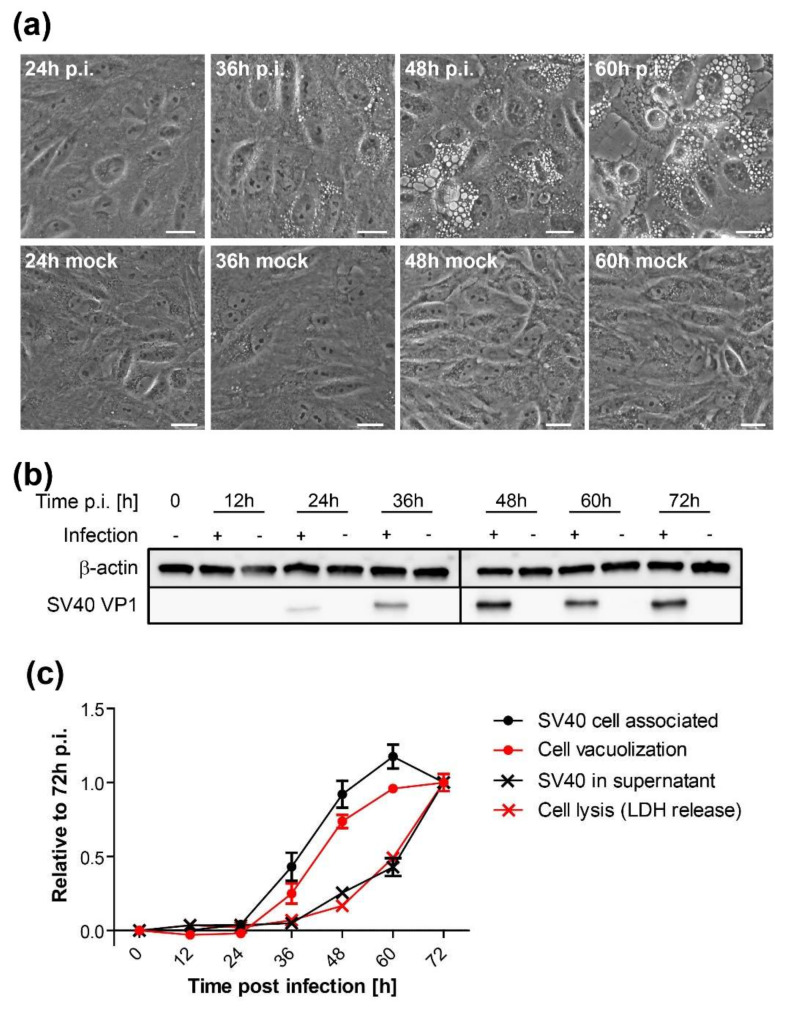
SV40-induced vacuole formation precedes cell lysis and virus release: (**a**) Time series of vacuole formation after infection of CV-1 cells with wildtype SV40 at an MOI of 10 and mock-treated CV-1 cells. Vacuolization was monitored by bright-field microscopy at the indicated h.p.i. Scale bar, 20 µm; (**b**) Western blot analysis of VP1 and actin expression in mock- and SV40-infected CV-1 cells at the indicated times p.i. Mock-infected cells at each time point were used as control; (**c**) Quantitation of vacuolization, cell-associated SV40, cell lysis, and SV40 release over the time course of an SV40 infection: CV-1 cells were infected and the number of vacuolated cells at different time points after infection was quantified from bright-field images as depicted in (**a**). A minimum of 200 cells per sample and three independent experiments were analyzed. Relative infectious units of cell-associated SV40 and released SV40 were quantified from cell lysates and supernatant, respectively, by titration onto CV-1 cells and flow cytometry analysis of large T antigen. CV-1 cell lysis was determined by quantitation of lactate dehydrogenase (LDH) in the supernatant using a colorimetric enzymatic assay, in which differences in the optical density between SV40-infected cells and mock-infected controls were determined. All values are displayed relative to 72 h.p.i. Mean ± SEM from three independent experiments are shown.

To study the temporal relationship between SV40-induced vacuolization and progression of the virus life cycle, CV-1 cells were infected with wildtype SV40 at an MOI 10 and vacuolization was monitored every 12 h by bright-field microscopy. Small vacuoles first appeared at 36 h.p.i., with a pronounced vacuolization evident by 48 h.p.i. (Figure 4A,C). Vacuolization reached a plateau at around 60 h.p.i. As expected, the appearance of vacuoles correlated with the expression of VP1, which became prominent around 36 h.p.i. as assessed by immunoblotting and increased at later times (Figure 4B). No changes in vacuolization were observed in mock-infected cells at any time point (Figure 4A).

To examine the temporal relationship between virus production and vacuole formation, we measured cell-associated and released infectious SV40. We infected CV-1 cells with SV40 at an MOI of 10, and at various times p.i., the supernatant was collected as a source of released virus. At the same time points, the cells were lysed by freeze-thawing as a source of cell-associated virus. Infectious virus in both samples was quantified by infecting naïve CV-1 cells and enumerating large T antigen-positive cells by flow cytometry 24 h later. As shown in Figure 4C, the timing and extent of vacuolization (red dots) were similar to the production of cell-associated virus, which first appeared at 36 h.p.i. (black dots). In contrast, significant amounts of infectious SV40 in the supernatant were first detected 48 h.p.i. and continuously increased until the end of the observation period at 72 h.p.i. (black Xs). Finally, we assessed cell lysis by measuring the release of the intracellular enzyme lactate dehydrogenase (LDH) into the supernatant. LDH levels in the supernatant coincided with released SV40 (red Xs). Thus, cell lysis and release of significant amounts of virus lag approximately 12 to 24 h behind intracellular virus production and vacuolization.

### 3.4. SV40 Induces MAPK Signaling Late During Infection

**Figure 5:**
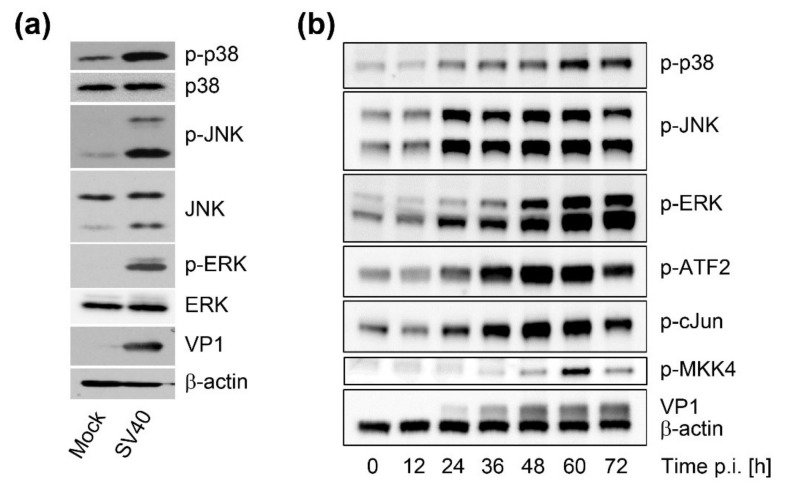
SV40 infection activates intracellular signaling pathways at late times after infection. (**a**) Western blot analysis of phosphorylated and total p38, JNK, and ERK in mock-infected or SV40-infected CV-1 cells 48 h.p.i.: VP1 and beta-actin expression are shown as controls; (**b**) Western blot analysis of CV-1 cells over the time course of SV40 infection: Samples harvested at the indicated h.p.i. were analyzed for phosphorylated p38, JNK, ERK, MKK4, ATF2, and c-Jun as well as for beta-actin and VP1 expression.

Infection by polyomaviruses at a high MOI transiently activates cellular mitogenic signaling pathways, including the MAP kinase pathways, which are triggered by Ras activation [9,10,11,12]. To determine whether the MAP kinase pathway was activated at late times after SV40 infection, when vacuoles typically form, we used phospho-site-specific antibodies and western blotting to examine phosphorylation of the signaling proteins JNK, p38, and ERK. At 48 h.p.i., we observed pronounced phosphorylation of JNK (Thr183/Tyr185), p38 (Thr180/Tyr182), and ERK (Thr202/Tyr204) compared to mock-infected cells, with little difference in the total amount of these proteins, indicating broad activation of these signaling pathways in response to SV40 infection (Figure 5A). We also examined the time course of MAP kinase activation by analyzing a series of time points beginning at 12 h.p.i., long after the acute phase of transient signaling induced by the addition of virus and fresh growth medium (data not shown). This analysis revealed progressive phosphorylation of JNK, ERK, and p38 beginning as early as 24 h.p.i. (Figure 5B). Activation of signaling pathways at cell membranes can lead to JNK1/2 phosphorylation through the action of MKK4, a membrane-proximal kinase. To determine the phosphorylation status of MKK4 during SV40 infection, we conducted western blot analysis of MKK4 phosphorylation at serine 257 and threonine 261. This analysis revealed the presence of phospho-MKK4 by 48 h.p.i. (Figure 5B). Although MKK4 phosphorylation was detected at later times than JNK phosphorylation, we believe that this is due to the lower sensitivity of the phospho-MKK4 antibody. After phosphorylation, activated MAP kinases translocate into the nucleus and regulate gene expression by phosphorylating transcription factors such as ATF-2 and c-Jun. Consistent with activation of MAP kinase signaling, ATF-2 and c-Jun were phosphorylated late during SV40 infection, when VP1 expression became abundant (Figure 5B).

### 3.5. JNK, MKK4, and Rac1 are Required for Vacuolization and SV40 Release

**Figure 6:**
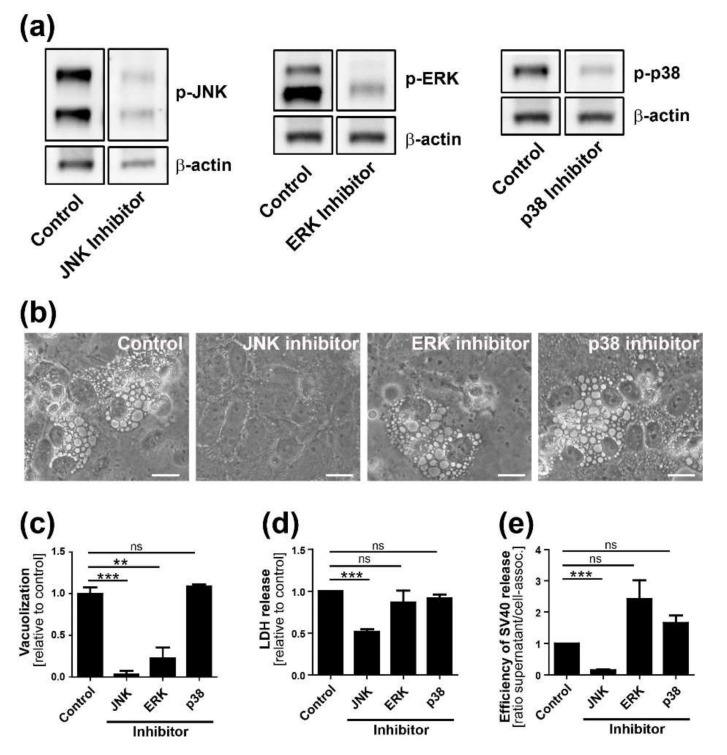
MAP kinase components are required for efficient vacuolization, cell lysis, and virus release. (**a**) Inhibitors SP600125, Selumetinib, and SB203580 inhibit SV40-induced phosphorylation of JNK, ERK, and p38, respectively. CV-1 cells were infected at an MOI of 10. Inhibitor treatment was started at 12 h.p.i., and immunoblotting was performed on extracts prepared 48 h.p.i; (**b**) Bright-field images of CV-1 cells 48 h.p.i. after infection with SV40: CV-1 cells were infected at an MOI of 10 and treated with inhibitors against JNK, ERK, and p38 or DMSO vehicle at 12 h.p.i.. Scale bar, 20 µm; (**c**) The number of vacuolated cells two days after infection was quantified and normalized to DMSO-treated control cells. *** *p* < 0.001 and ** *p* < 0.01; (**d**) Analysis of cell lysis two days postinfection with SV40 in CV-1 cells treated with inhibitors: LDH activity in the supernatant was measured. *** *p* < 0.001; (**e**) The ratio of released SV40 in supernatant versus cell-associated SV40 is shown. Data were normalized to DMSO-treated control infected cells. Quantitation of cell-associated SV40 and SV40 in the supernatant 48 h.p.i. of CV-1 cells treated with JNK, ERK, and p38 inhibitors is shown in Supplemental Figure S2. The mean values ± SEM from three independent experiments are shown. *** *p* < 0.001.

To determine the role of specific signaling pathways in vacuolization and SV40 release, we tested whether chemical and genetic inhibitors of signaling affected these processes. Starting at 12 h.p.i., CV-1 cells were treated with widely used and selective chemical inhibitors targeting JNK (SP600125), p38 (SB203580), and MEK (Selumetinib), a key component of the ERK pathway. Inhibitory activity was confirmed by western blot analysis showing reduced phosphorylation of the target protein at 48 h.p.i. (Figure 6A). Vacuolization of cells treated with the inhibitors was examined two days p.i.. Strikingly, the JNK inhibitor SP600125 completely blocked vacuole formation, whereas p38 inhibition had no effect compared to the control infected cells treated with vehicle alone (Figure 6B,C). The ERK pathway inhibitor caused a significant reduction in vacuolization, but strong cytotoxic effects were observed during treatment of noninfected cells with this compound (Supplemental Figure S2A). We conclude that the lack of vacuoles in ERK-inhibited cells is likely a consequence of accelerated cell death and detachment of cells rather than a true suppression of vacuolization. 

Inhibition of JNK also inhibited SV40-induced cell lysis by 50% (Figure 6D) and caused a 6-fold reduction of released SV40 compared to cell-associated virus (Figure 6E and Figure S2B,C). In contrast, treatment of infected cells with the MEK or p38 inhibitor did not reduce cell lysis or SV40 release compared to vehicle-treated control infected cells. Treatment of cells with inhibitors did not interfere with early steps of SV40 infection as assessed by flow cytometry for large T antigen expression (Supplemental Figure S2D), suggesting that the JNK signaling pathway is specifically required late in infection for vacuolization, cell lysis, and efficient virus release.

**Figure 7:**
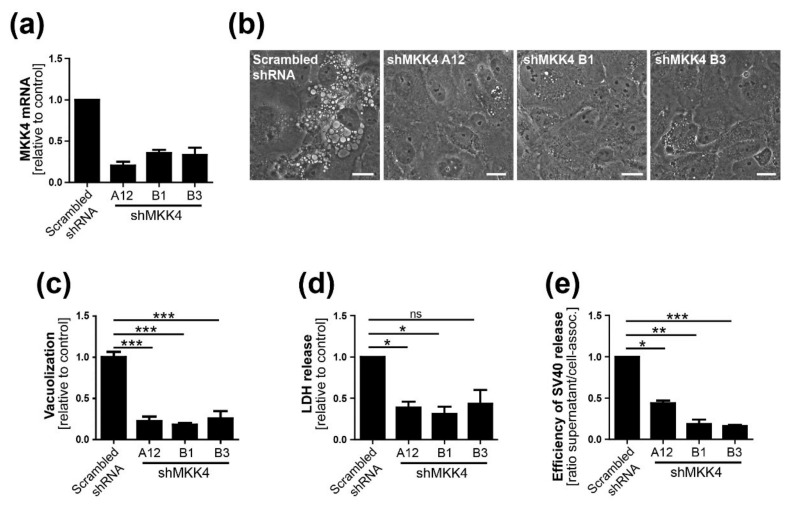
MKK4 is required for efficient SV40-induced vacuolization, cell lysis, and virus release. (**a**) qPCR analysis of MKK4 mRNA expression levels in CV-1 cells stably expressing three different shRNAs targeting MKK4 (A12, B1, and B3): levels of mRNA were normalized to mRNA in control cells expressing scrambled shRNA; (**b**) Images of SV40-infected control and MKK4 knockdown cells: CV-1 cells stably expressing scrambled control shRNA or MKK4 A12, B1, or B3 shRNA were infected with SV40, and vacuole formation was monitored by bright-field microscopy at 48 h.p.i. Scale bar, 20 µm; (**c**) The number of vacuolated cells as in panel B was quantified and normalized to scrambled shRNA control cells. A minimum of 200 cells per sample were analyzed. The mean values ± SEM from three independent experiments are shown. *** *p* < 0.001; (**d**) CV-1 cells expressing three different MKK4 shRNAs were infected with SV40, and the supernatants were analyzed for LDH release at 48 h.p.i. * *p* < 0.05; (**e**) The ratio of released SV40 in supernatant versus cell-associated SV40 is shown. (Quantitation of cell-associated SV40 and SV40 in supernatant of MKK4 knockdown cells 48 h.p.i. is shown in Supplemental Figure S3A,B) Data were normalized to scrambled shRNA control infected cells. *** *p* < 0.001, ** *p* < 0.01, and * *p* < 0.05.

To assess the role of MKK4 in SV40-induced vacuolization and virus release, we used three shRNAs with different MKK4 target sequences to generate CV-1 cells with stable MKK4 knockdown (Figure 7A). MKK4 knockdown by each of these shRNAs reduced SV40-induced cell vacuolization (Figure 7B,C) and cell lysis (Figure 7D) compared to scrambled shRNA control. Notably, MKK4 knockdown also caused a significant reduction of released SV40 virions compared to cell-associated SV40 (Figure 7E; Supplemental Figure S3A,B). MKK4 knockdown did not affect the efficiency of SV40 infection as assessed by intracellular virus production (Supplemental Figure S3A) and by flow cytometry and western blotting for large T antigen and VP1 expression (Supplemental Figure S3C,D).

**Figure 8:**
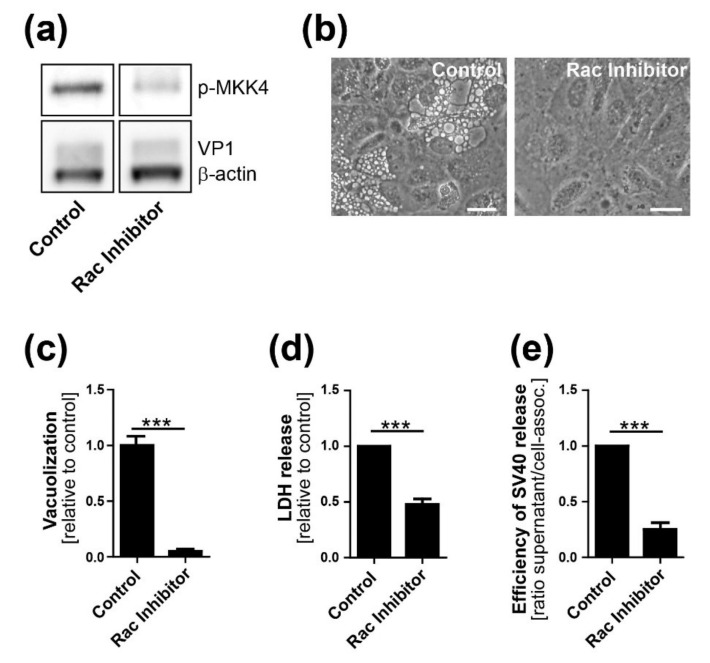
Rac1 activity is required for efficient SV40-induced vacuole formation, cell lysis, and virus release. CV-1 cells were infected with SV40 at at MOI of 10. At 12 h.p.i., the Rac1 inhibitor EHT1864 was added and cells were analyzed at 48 h.p.i. (**a**) Western blot analysis of MKK4 phosphorylation in SV40-infected CV-1 cells in the presence and absence of Rac1 inhibitor: a representative western blot of 4 independent experiments is shown; (**b**) Infected CV-1 cells were treated with EHT1864 or DMSO control and photographed by bright-field microscopy. Scale bar, 25 µm; (**c**) Quantitation of vacuolated CV-1 cells after SV40 infection as described in Figure 6D. *** *p* < 0.001; (**d**) Infected CV-1 cells were treated with EHT1864. At 48 h.p.i., LDH released in the supernatant was determined. *** *p* < 0.001; (**e**) The ratio of released SV40 in supernatant versus cell-associated SV40 is shown. To quantify virus release, infectious units of SV40 in cell lysates and supernatants were analyzed by infection and flow cytometry, as described in Figure 2D–F. Quantitation of cell-associated SV40 and SV40 in the supernatant in Rac1 inhibitor-treated CV-1 cells at 48 h.p.i. is shown in Supplemental Figure S4A,B. *** *p* < 0.001.

JNK and MKK4 signaling is required for methuosis, a Ras-dependent form of cell death characterized by extensive vacuolization [28]. The small GTPase Rac1 has been identified as a mediator of methuosis upstream of MKK4 and JNK. Therefore, we tested the role of Rac1 in SV40-induced vacuolization, cell lysis, and virus release. Treatment of CV-1 cells at 12 h.p.i. with the specific Rac1 inhibitor EHT1864 blocked downstream phosphorylation of MKK4 compared to vehicle-treated infected cells, confirming inhibition of the Rac1-MKK4-JNK signaling cascade (Figure 8A). Rac1 inhibition also significantly reduced cell vacuolization (Figure 8B,C), cell lysis (Figure 8D), and release of infectious SV40 (Figure 8E and Supplemental Figure S4A,B). Production of intracellular virus and SV40 infectivity were not inhibited by EHT1864 (Supplemental Figure S4A,C). Taken together, these results support a model in which a Ras-dependent cell signaling cascade involving Rac1, MKK4, and JNK induces extensive cell vacuolization, followed by cell lysis and SV40 release.

## 4. Discussion

Activation of cellular signaling pathways is important during various steps in virus life cycles. For example, virus binding to its surface receptor often triggers signaling cascades that facilitate virus entry and replication. We previously reported that intracellular SV40 VP1 expression alone does not induce vacuolization and that SV40 particles and VP1 are absent from the vacuolar lumen in infected CV-1 cells or in CV-1 cells undergoing vacuolization in response to acute treatment with L1 pentamers [19]. These findings suggest that vacuolization is the result of a signaling cascade triggered at the cell surface. In this report, we show that, in addition to signaling occurring early in infection, SV40 also induces the MAPK signaling cascade during the late stage of infection when large amounts of VP1 accumulate to support vacuolization and efficient virus release.

Several findings reported here suggest that progeny SV40 particles bind to GM1 at the plasma membrane and trigger GM1-dependent Ras activation and vacuolization to support further virus release. Live-cell microscopy of infected CV-1 cells revealed that SV40-induced vacuoles are positive for endosomal marker proteins (Rab5, Rab7, and LAMP-1) and display a dynamic endocytic nature, resembling LAMP-1-positive cytoplasmic vacuolation induced by Ras activation (HRas GV12) [25,29]. We also show that VP1 co-localizes with GM1 and Ras at the limiting membrane of SV40-induced vacuoles arising late in infection. Most importantly, we showed that dominant-negative Ras S17N or inhibition of JNK signaling inhibits vacuole formation. Overall, we hypothesize that, in response to SV40-induced clustering of GM1, Ras is activated and triggers MAPK signaling through Rac1-MKK4-JNK, which results in vacuolization and subsequent cell lysis and virus release. Mechanistic details of how and where VP1 interacts with GM1 to trigger downstream signaling pathways will require additional studies.

Previously published work is consistent with this model. GM1 clustering has been reported to modify active HRas distribution in membrane nanodomains [30]. Furthermore, overexpression of constitutively active HRas (HRas G12V) leads to extensive vacuolization in glioblastoma cells, with Ras localized at the limiting membrane of cytoplasmic vacuoles [25]. Like vacuoles formed during SV40 infection, HRas-induced vacuole formation in glioblastoma cells was independent of ERK signaling [25] and blocked by chemical inhibition of Rac1 [31]. Furthermore, in both ras-activated glioblastoma cells and SV40-infected cells, vacuoles contain endosomal markers such as Lamp-1 and undergo extracellular fluid uptake resembling macropinocytosis [24,25,32].

Although Ras and VP1 co-localize in vacuolar membranes (Figure 1C), we have not determined the cellular compartment where GM1-dependent Ras activation occurs during SV40 infection. GM1 can localize to several different membrane compartments including the plasma membrane, Golgi network, and the ER, and the ceramide structure of GM1 influences its trafficking into various compartments [33]. Similarly, Ras localizes not only to the plasma membrane but also to internal membranes such as the limiting membranes of endosomes, the ER, and the Golgi network [34]. FRET-based assays demonstrated that EGF stimulation changed the distribution of endogenous active Ras from the plasma membrane to endosomal-like intracellular vesicles, the Golgi network, and the ER [35]. Moreover, the subcellular localization of active Ras influences signaling through downstream effector pathways [36]. Thus, SV40 may activate Ras in various cellular compartments at different steps during the viral life cycle, with different biological consequences.

Time course analysis of SV40-induced late cellular signaling revealed that it precedes vacuolization, cell lysis, and virus release. Active phosphorylated forms of JNK were detected 24–36 h.p.i., coincident with VP1 expression, followed by phosphorylation of nuclear transcription factors. Unlike the acute transient signaling induced by virus cell binding or growth factor receptor signaling upon the addition of fresh growth medium, signaling in this late phase of infection is sustained. Activation of cell signaling preceded vacuole formation, with the first vacuoles emerging at 36–48 h.p.i., prior to cell lysis and virus release. Although other cellular kinases such as p38 and ERK were also activated during SV40 infection, inhibitor experiments revealed that only the JNK pathway is essential for vacuolization, cell lysis, and efficient virus release. Importantly, inhibition of JNK, Ras, and MKK4 activity or expression did not interfere with intracellular replication of SV40, so the impaired late events do not merely reflect an early replication block. This is consistent with an earlier published report that MAPK/ERK signaling is not required for early response to SV40 infection [12] and with a recent genome-wide analysis of kinases that contribute to SV40 endocytosis, which did not detect a requirement for MAPK signaling [13]. We conclude that Ras-Rac1-MKK4-JNK signaling is essential late during SV40 infection for vacuolization and cell death leading to virus release, although a contribution of other signaling pathway components such as MKK7 cannot be ruled out.

In agreement with previous studies [37], we showed that infectious SV40 first appears at low levels in the supernatant as early as 36 h.p.i. The release of virus at this early time might occur due to the first cells that lyse or to virus released via a non-lytic process. We hypothesize that SV40 released from cells around this time binds to the plasma membrane of the same and neighboring infected cells and induces cell signaling, which in turn stimulates cell lysis and subsequent increased virus release from these cells, thus establishing a positive feedback loop that stimulates further signaling and virus release. This model is similar to our earlier analysis of vacuolization during SV40 infection [19], in which we proposed that the first released virus binds to cell surface GM1 and stimulates vacuole formation late during infection. We extend the model here to include virus release as well as vacuolization as a phenotype that can be acutely triggered late in infection by the first progeny virus released. Thus, the initial wave of released virus primes the infected cell population for more pronounced vacuolization and enhanced virus release.

To complete the virus life cycle, polyomavirus release depends on lysis of the infected cell. Whether cell death is the consequence of plasma membrane rupture resulting from extensive virus production or a regulated process depending on active cellular signaling was heretofore unclear. Here, we show that high levels of intracellular virus are not sufficient for efficient SV40 release and that cellular MAPK signaling is necessary for optimal cell lysis and virus release. This lytic process resembles methuosis, which, as noted above, is a Rac1-dependent cell death pathway displaying characteristic cellular vacuolization occurring after ectopic expression of oncogenic Ras [24,25,29].

Vacuolization and virus release are both facilitated by MAPK signaling, and vacuolization precedes virus release. If activation of the Ras-MAPK signaling cascade independently induces both vacuole formation and virus release, vacuolization is a convenient marker for the signaling events that foster efficient release. Alternatively, it is possible that vacuolization itself facilitates subsequent cell death and enhanced virus release.

In addition to the role of cellular signaling, virus-encoded proteins could also be involved in cell lysis and SV40 release. VP4 is a late SV40 protein previously reported to function as a viroporin to support virus release [38]. VP4 was also reported to disrupt membranes when ectopically added to red blood cells, liposomes, or Cos-7 cells [38]. However, more recent studies did not confirm the lytic activity of VP4 during SV40 infection [16]. In the context of SV40 replication, the expression of VP1 alone, without VP2 and VP3, leads to cell lysis and the release of viral particles, suggesting that lytic activity is mediated via VP1, likely through activation of a cellular program as reported here [14,16].

Our results raise the possibility that JNK and MAPK pathway inhibitors may have a role in treating polyomavirus infections by decelerating virus propagation and spread within the host by reducing virus release. Such a scenario would presumably provide a protective effect in affected tissues while immune reconstitution is underway [39]. Because of the long replication cycle of human polyomaviruses and difficulties in synchronizing infection, late events are difficult to study. Nevertheless, further studies of the human pathogenic polyomaviruses may reveal that they are also affected by signaling programs late during infection. Although human polyomaviruses bind only weakly to GM1 [39], a variety of stimuli can activate the signaling elements described here. Thus, analysis of late events of human polyomavirus infection might establish that this or overlapping signaling pathways are viable therapeutic targets.

## Supplementary Materials

The following are available online at https://www.mdpi.com/1999-4915/12/10/1128/s1, Figures S1–S4 and Movies S1 and S2.
