# Supplementary material for: SV40 Polyomavirus Activates the Ras-MAPK Signaling Pathway for Vacuolization, Cell Death, and Virus Release
Source: Viruses. 2020 Oct 5;12(10):1128. doi: 10.3390/v12101128 (PMC7650553; doi:10.3390/v12101128)
Supplement: Supplementary file 1 [file viruses-12-01128-s001.zip › Supplemental Figures + legends.pdf]

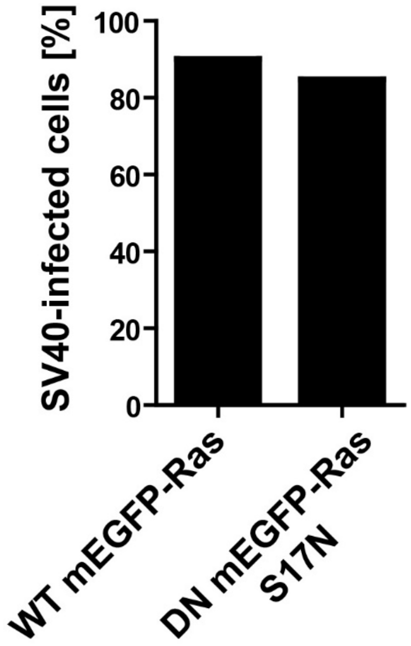

Supplemental Figure S1. Expression of dominant-negative Ras does not affect infection levels of CV-1 cells. CV-1 cells were transfected with wild-type mEGFP-HRas (WT) or dominant-negative mEGFP-HRas S17N (DN), respectively. Twelve hours p.i., cells were infected with SV40 at MOI of 10. At 24 h.p.i., cells were harvested and stained for large T antigen. Cells were analyzed by flow cytometry for GFP and T antigen fluorescence. The fraction of infected cells in the GFP^+ population is shown.

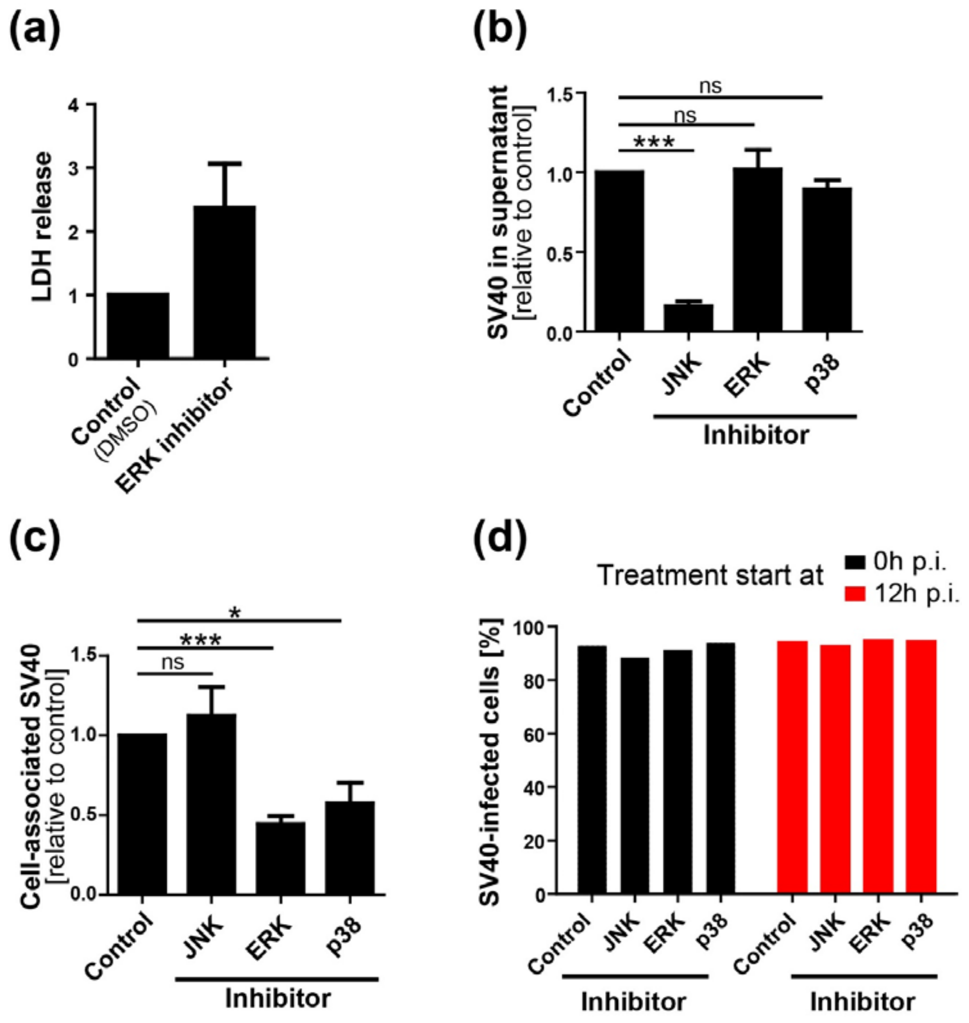

Supplemental Figure S2. Effect of MAPK inhibitors on SV40 replication and cell viability. (**a**) ERK inhibitor reduces cell viability as assessed by LDH release. Mock-infected CV-1 cells were treated with ERK inhibitor or vehicle alone for 36 h and read out for LDH activity in the cell supernatant. Depicted is the mean of three independent experiments +/- SEM. **(b)** Treatment with JNK Inhibitor SP600125 reduces release of SV40 from infected cells. CV-1 cells were infected at MOI of 10. Inhibitor treatment was started at 12 h.p.i. and infectious units in the supernatant were quantified at 48 h.p.i. (**c**) Treatment with JNK inhibitor does not affect virus production. CV-1 cells were treated as in (b). Infectious units were quantified in cells at 48 h.p.i. **(d)** Inhibitor treatment of CV-1 cells does not influence SV40 infectivity. CV-1 cells were infected at MOI of 10 and treated with inhibitors against JNK, ERK, and p38 or DMSO vehicle at the time of infection (0 h.p.i. – black bars, 12 h.p.i.- red bars). At 24 h.p.i., cells were harvested, stained for expression of large T antigen and read out by flow cytometry.

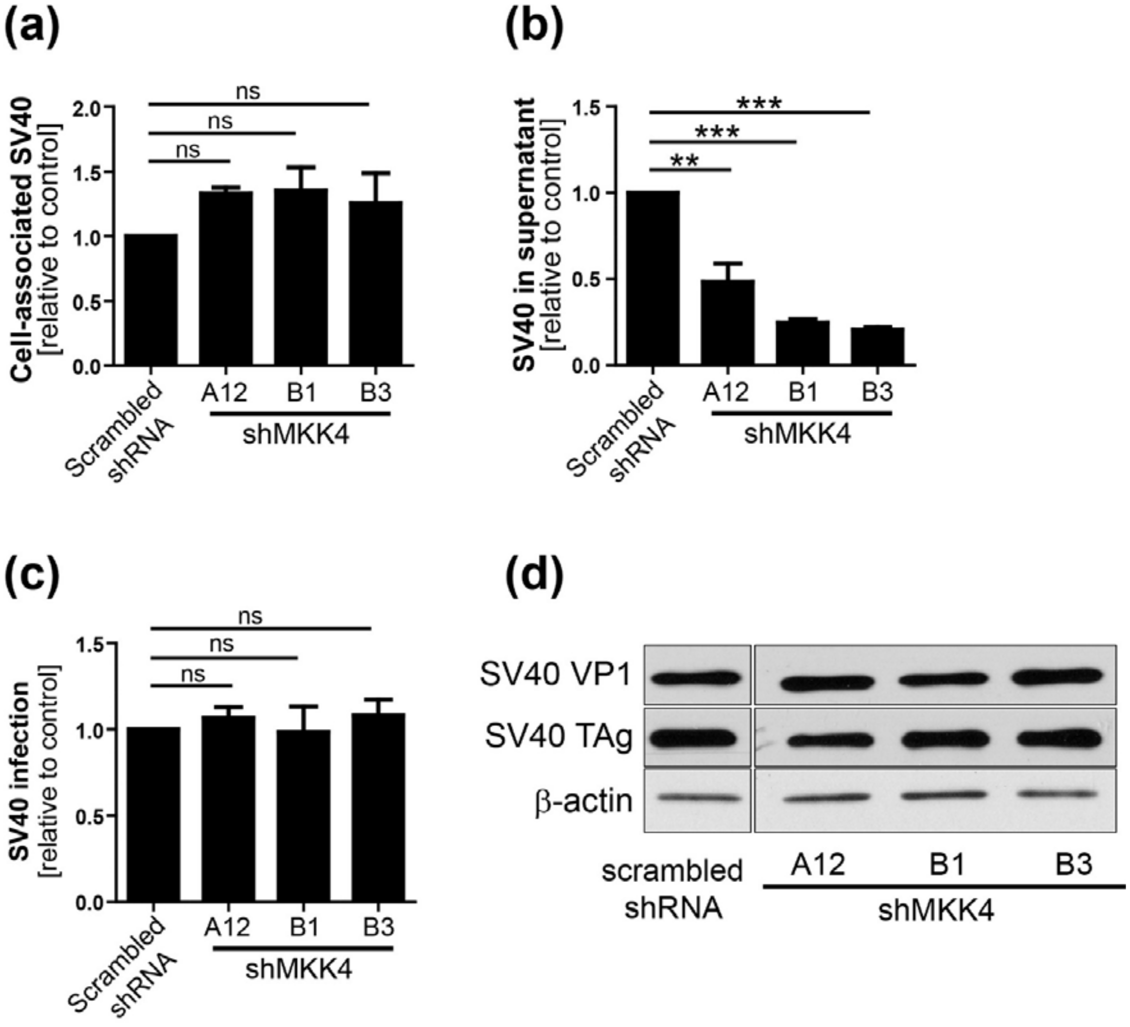

Supplemental Figure S3. MKK4 knock-down reduces SV40 release while maintaining infection levels and virus replication. (a) MKK4 knockdown does not affect virus production. CV-1 cells expressing shRNAs to MKK4 or scrambled shRNA were infected with SV40 at MOI of 10. At 48 h.p.i., cells were harvested for quantitation of infectious units. **(b)** MKK4 is required for efficient virus release. Experiments were set up as in (a). At 48 h.p.i., infectious units were quantified in the supernatant. **(c, d)** MKK4 knockdown does not influence SV40 infectivity or replication. Experiments were set up as described in (a). (c) At 24 h.p.i. infected cells were harvested and subjected to large T antigen staining for determination of infection levels. Depicted is the mean of three experiments +/-SEM. (d) At 48 h.p.i., expression levels of the early protein large T antigen and the late protein VP1 were determined by immunoblotting.

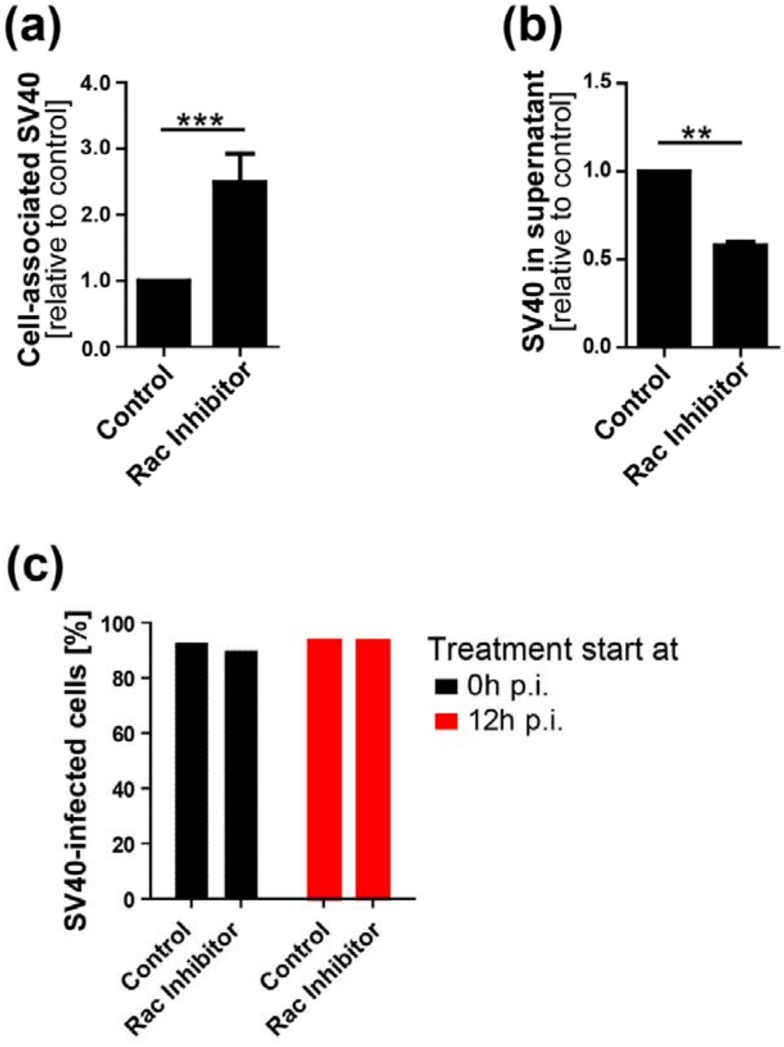

Supplemental Figure S4. Effect of Rac1 inhibitor EHT1864 on SV40 replication. (a) Rac1 inhibition increases levels of cell-associated virus. CV-1 cells were infected at MOI of 10. Inhibitor treatment was started at 12 h.p.i. and cell-associated infectious units were quantified at 48 h.p.i. **(b)** Treatment with Rac1 Inhibitor EHT1864 reduces release of SV40 from infected cells. CV-1 cells were treated as in (a). Infectious units in the supernatant were quantified at 48 h.p.i.. (**c**) Inhibitor treatment of CV-1 cells does not influence SV40 infectivity. CV-1 cells were infected at MOI of 10 and treated with Rac1 inhibitor EHT1864 or DMSO vehicle at the time of infection (0 h.p.i. – black bars) or 12 h.p.i (red bars). At 24 h.p.i., cells were harvested, stained for expression of large T antigen and read out by flow cytometry.
